# A Few Suggestions on the Treatment of Diseases of the Eye and Ear

**Published:** 1890-04

**Authors:** James L. Minor

**Affiliations:** Memphis, Tenn.; Late Attending Surgeon to the New York City Hospitals on Randall’s Island


### Selections.

### A FEW SUGGESTIONS ON THE TREATMENT OF DISEASES OF THE EYE AND EAR.

JAMES L. MINOR, M. D., Memphis, Tenn.
Late Attending Surgeon to the New York City Hospitals on Randall's Island.

I am prompted to make these suggestions by a knowledge of the fact that by far the larger number of patients with eye or ear disease, fall under the care of the general practitioner, who, in student days, found these subjects not only dull and uninteresting, but complicated—hence he has simply attempted to get the general principles, without a thought of obtaining a thorough mastery of the subject—a thing difficult of accomplishment, when professor and text-book both dwell so much upon details. I felt this keenly myself when in general practice, and have heard frequent reference to, and seen many illustrations of it since I devoted special study to these diseases. I shall, in a general way, and briefly, attempt to give simply the treatment of those diseases most frequently seen, by suggesting the use of a few remedies which will be useful to the greatest variety, and hurtful to but few, or none, of those diseases liable to be mistaken for one another.

The most frequently observed disease of the eye, is *catarrhal conjunctivitis*, or ordinary "cold" of the eyes, which with simple cleanliness is, in many instances, a self-limited disease. A cure can be hastened, however, by local applications; and in the choice of these, preference should be given to the milder forms of eye-washes, for they are in nine cases out of ten equally as efficacious as the stronger applications. They are not unpleasant to the eye, and can do no harm. If inflammation of the eye (conjunctiva) assumes an active type, there is apt to be hyperæmia of the iris, which readily passes into inflammation of that structure, under the influence of strong applications to the lids or globe; and the same may be said of the cornea—hence the safety of mild remedies, and the danger of strong ones. Either of the following prescriptions will meet the indications of a mild eye-wash:

1. A solution of common salt (grs. x ad ℥i).

2. A saturated solution of boracic acid (grs. xv ad ℥i).

3. ℞ Sodii biboratis ℈iv, aquæ camphoræ, aquæ, aa ℥ii. M.

4. ℞ Zinci sulphatis gr. i, acidi boracici ʒi, aquæ ℥iv M.

These may be freely applied to the eye, without fear of harm. As examples of what I consider the stronger eye-washes, I may cite solutions of copper, of zinc, of alum, of nitrate of silver, of acetate of lead, as strong as five or ten grains to the ounce.

The next disease of the eye in order of frequency is inflammation of the cornea, or *keratitis*, which is sometimes associated with the catarrhal ophthalmia just considered, and in many instances the casual observer will place the two diseases in the same category. And yet, the strong applications, which the inflamed conjunctiva would stand, not only with impunity, but with marked benefit, might seriously endanger an eye affected with keratitis. Here treatment *must be mild*, if safety of the eye is consulted. Any one of the prescriptions which I have suggested can be used with benefit and without danger, and it is well to use in addition some soothing application, as ℞ Atropiæ sulphatis, cocain. muriat. aa gr. ii, aquæ ℥i. M. Sig. Put two drops in the eye three times a day.

Another disease of the eye—inflammation of the iris, or *iritis*—is often seen, and it, too, has so many symptoms in common with the diseases already considered that it is liable to be mistaken for either. Here all the usual eye-washes are objectionable—their danger increasing with their astringency. The prescriptions which I have given, are at least open to this objection—and while they can do no good, they can hardly be considered as dangerous. The sheet-anchor here, is atropia, which can be advantageously combined with cocaine, four grains each, of cocaine and atropine to the ounce of water. This should be used sufficiently often to keep the pupil dilated, and until the eye is free from redness. Attention should of course be given to the general health in every instance. Either the syphilitic taint or the rheumatic habit will usually be found with iritis. Whenever the eyeball is red and inflamed, with dread of light, or haziness of the cornea or a contracted or sluggish pupil, rely upon atropine and cocaine, and use no stronger application than a solution of boracic acid. When an absence of these symptoms shows that the trouble is in the lids, stronger applications are admissible.

A few points about diseases of the ear, and I shall cease.

Ordinary *earache*, is an inflammation of the middle ear, and when the process goes on to pus-formation, an abscess on the inner side of the drum membrane is the result. The pressure from the pent-up pus causes a rupture of the drum, through which the matter escapes. This is often an end of the trouble, but frequently the inflammation continues—the opening in the drum remains—disease of the bones of the ear develops, and a more or less continuous discharge, an otorrhœa, is the result. If hot water be liberally and frequently injected into the ear through a douche, the inflammation will usually be stopped and a cure effected. Two or three drops of hot laudanum dropped into the ear will often accomplish the same purpose. After the discharge appears it can ordinarily be checked by syringing the ear often enough to keep it clean, with warm water containing boracic acid in the proportion of fifteen grains to the ounce; and, if, in addition to the syringing, a little pulverized boracic acid is blown into the ear through a quill or tube, after the ear is cleansed, this treatment will usually suffice to cure an otorrhœa.

In removing plugs of wax, or foreign bodies which have gained access to the ear, it is better to rely upon warm water and a syringe, than to resort to instruments. It is not only easier but more efficacious and safer. With the most delicate touch, it is as difficult to handle an instrument with precision in the deep and small cavity of the ear, as it is to avoid inflicting injury to these delicate parts which may be more serious than the trouble for which it was undertaken.—*Memphis Journal Medical Sciences.*

